# Length-Based Assessment of Coral Reef Fish Populations in the Main and Northwestern Hawaiian Islands

**DOI:** 10.1371/journal.pone.0133960

**Published:** 2015-08-12

**Authors:** Marc O. Nadon, Jerald S. Ault, Ivor D. Williams, Steven G. Smith, Gerard T. DiNardo

**Affiliations:** 1 University of Miami, Rosenstiel School of Marine and Atmospheric Science, 4600 Rickenbacker Causeway, Miami, FL, 33149, United States of America; 2 Joint Institute for Marine and Atmospheric Research, 1000 Pope Road, Honolulu, HI, 96822 United States of America; 3 NOAA Fisheries, Pacific Islands Fisheries Science Center, 1845 Wasp Boulevard, Honolulu, HI, 96818, United States of America; Leibniz Center for Tropical Marine Ecology, GERMANY

## Abstract

The coral reef fish community of Hawaii is composed of hundreds of species, supports a multimillion dollar fishing and tourism industry, and is of great cultural importance to the local population. However, a major stock assessment of Hawaiian coral reef fish populations has not yet been conducted. Here we used the robust indicator variable “average length in the exploited phase of the population ($\bar{L}$)”, estimated from size composition data from commercial fisheries trip reports and fishery-independent diver surveys, to evaluate exploitation rates for 19 Hawaiian reef fishes. By and large, the average lengths obtained from diver surveys agreed well with those from commercial data. We used the estimated exploitation rates coupled with life history parameters synthesized from the literature to parameterize a numerical population model and generate stock sustainability metrics such as spawning potential ratios (SPR). We found good agreement between predicted average lengths in an unfished population (from our population model) and those observed from diver surveys in the largely unexploited Northwestern Hawaiian Islands. Of 19 exploited reef fish species assessed in the main Hawaiian Islands, 9 had SPRs close to or below the 30% overfishing threshold. In general, longer-lived species such as surgeonfishes, the redlip parrotfish (*Scarus rubroviolaceus*), and the gray snapper (*Aprion virescens*) had the lowest SPRs, while short-lived species such as goatfishes and jacks, as well as two invasive species (*Lutjanus kasmira* and *Cephalopholis argus*), had SPRs above the 30% threshold.

## Introduction

The Hawaiian coral reef ecosystem is inhabited by hundreds of reef fishes and macroinvertebrates and supports multimillion-dollar fishing and tourism industries [1]. The principal targeted fish taxa include larger jacks and snappers, but also smaller reef-associated families such as surgeonfishes, goatfishes, squirrelfishes, and parrotfishes [2]. The coral reef fishery includes a mix of nearshore recreational/subsistence fishing combined with a commercial sector. The fishery is mostly shore-based fishers using a range of gears that includes spears, hook-and-line, traps, and small gill and cast nets. Commercial marine landings in Hawaii are dominated (>80% of catches) by coastal-pelagic species [3], but also include the smaller families listed above. The commercial fishery comprises an important aquarium-trade component, in which many species are taken, but which is primarily focused on small-bodied surgeonfishes. Although the direct monetary value of the near-shore fishery is only 10–20% of the pelagic fishery [4], it is both culturally and socially important. Recreational catches have been estimated to exceed the commercial sector, with nearly a third of all households participating [2,5–7].

Observational studies have shown drastic differences in Hawaiian reef fish abundance along inferred fishing intensity gradients [8–10]. For example, a recent study showed that snapper and parrotfish biomasses are about 3–4 times higher in the unpopulated and remote Northwestern Hawaiian Islands than in the populated main Hawaiian Islands [11]. However, no formal quantitative stock assessment analysis has been conducted to date for individual species. This is mainly due to a lack of demographic data, time-series of age- or size-structured catches by species, and associated fishing effort [12–14]. This relatively “data poor” situation has been steadily improving in the Hawaiian Archipelago where implementation of large-scale fishery-independent coral reef fish surveys [9], along with increased efforts on life history and demographic research, now allows the possibility of more in-depth assessments of the sustainability of coral reef fish fisheries.

The general goal of stock assessment is to use statistical and mathematical models to make quantitative predictions about the response of fish populations to exploitation and alternative management strategies [15]. These usually aim at optimizing yield while maintaining the reproductive capacity of a stock at a safe level [12,14]. In this paper we employed a length-based approach previously used to assess reef fish stocks in southern Florida and Puerto Rico [13,16] to conduct the first major stock assessment of the exploited coral reef fishes in Hawaii. Two other recent, but limited, assessment efforts [17,18], restricted to family level analyses, relied on incomplete commercial catch record time-series that did not capture the dominant recreational catches [6]. The length-based approach used here avoids relying on this incomplete dataset and instead used size composition data gathered from a range of sources to generate species-level assessments. Our first step was to estimate stock exploitation rates using a robust and straight-forward length-based total mortality assessment method suitable for the data-poor situation typical of coral reef fisheries [19]. The principal data used were size-composition information from both fishery-independent and-dependent sources. We combined these mortality rates with life history parameters (i.e., length-at-maturity, growth rates, and longevity) in a numerical population model to compute sustainability benchmarks. As an aid to fishery managers, we evaluated species-specific benchmarks with respect to resource sustainability standards. Finally, the availability of an extensive fishery-independent dataset from the NWHI, a large relatively pristine area, allowed us to test and validate the predictions of our stock assessment models and parameters.

## Methods

### Study area

**Fig 1:**
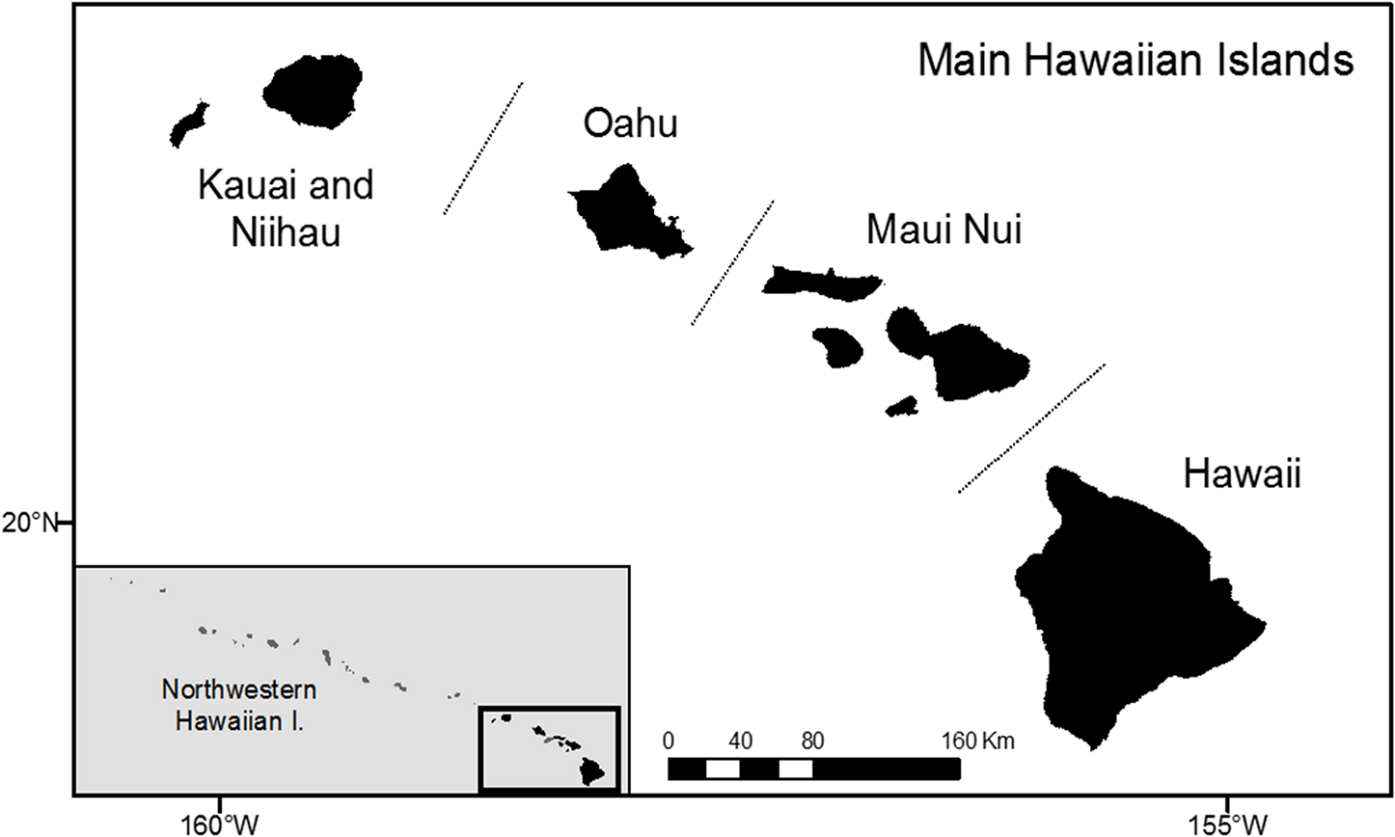
Map of the Hawaiian Archipelago. The four subregions of the main Hawaiian Islands used in the analyses are delineated by dashed lines (inset shows the two principal regions). This map was prepared in ArcGIS 10 and contains open access layers.

The Hawaiian Archipelago extends for 2600 km along a SE-NW axis from 19°N, 155°W to 28°N, 178°W (Fig 1). The archipelago, consisting of 18 islands and atolls, is typically divided into two broad regions: (1) the inhabited main Hawaiian Islands (MHI); and, (2) the sparsely inhabited Northwestern Hawaiian Islands (NWHI). The MHI is composed of 8 geologically young, high (4,205 m maximum elevation) volcanic islands that are densely human-populated (1.39 million persons; dbedt.hawaii.gov/census). In contrast, the NWHI are relatively low-lying (275 m max elevation) and sparsely inhabited (Table 1). The MHI were first settled by people around AD 1250, and reef fish communities in the MHI have been exploited to various degrees since that time [20]. The NWHI were never permanently settled; however, they were the focus of commercial fishing, particularly in the 19^th^ century [20]. Over the past century, the fishing fleet operating in the NWHI has remained relatively small at less than 12 vessels focused mainly on bottomfish (deepwater snappers) and lobsters [20]. In 20065, the NWHI and surrounding marine environment, including associated coral reefs and other resources, were designated as the Papahanaumokuakea Marine National Monument (PMNM) and protected under the co-management of several agencies of the U.S. federal government and the State of Hawaii.

**Table 1:** Information summary of the two principal regions of the Hawaiian Islands, including the four subregions of the main Hawaiian Islands.

Region	Human population (2010)	Reef area (km^2^)	Prop. of total reef in region	Pop’n per reef area (# km^-2^)	Channel width (km)^a^
Main Hawaiian Is.					
Hawaii	185,079	194	0.16	954	48
Maui Nui	154,950	373	0.31	415	48–42
Oahu	953,000	375	0.31	2541	42–116
Kauai-Niihau	65,819	266	0.22	247	116–220
Northwestern Hawaiian Is.	~100	1595	-	~ 0	220

^a^ Channel widths from east to west.

### Length-based mortality model

The principal stock assessment indicator variable used to quantify the population status for the community of Hawaiian reef fishes was average length ($\bar{L}$) in the exploited phase of the population. Average length is highly correlated with population size [21]. For exploited species, $\bar{L}$ directly reflects the rate of instantaneous fishing mortality *F* through alteration of the population composition [19,22]. As *F* increases, the probability of a fish reaching larger sizes decreases, and thus the mean of the exploited size frequency distribution decreases accordingly. Theoretically, the average length $\bar{L}$ is generally expressed as

$$\bar{L}=\frac{F\int_{a_{c}}^{a_{\lambda }}\;N\left(a\right)L\left(a\right)da}{F\int_{a_{c}}^{a_{\lambda }}\;N\left(a\right)da}\;,$$ (1)

where the exploitable phase is integrated from *a*_*c*_ (age at first capture) to *a*_*λ*_ (oldest age), *N(a)*is the abundance of age class *a*, *L(a)* is the expected length at age *a*, and *F* is the instantaneous fishing mortality rate.

A formula for estimating mortality rates using estimates of $\bar{L}$ was derived from Eq (1) by Ehrhardt and Ault [19]. The first step in this derivation was to substitute *L(a)* in Eq (1) with the von Bertalanffy growth function and *N(a)* with the exponential mortality model

$$N\left(a+\Delta a\right)=N\left(a\right)e^{-Z\Delta a}\;,$$ (2)

where *Z* is the total instantaneous mortality rate and *Δa* is the age interval, normally one year. Step two was to integrate and algebraically solve for *Z*,

$${\left(\frac{L_{\infty }-L_{\lambda }}{L_{\infty }-L_{c}}\right)}^{Z/K}=\frac{Z\left(L_{c}-\bar{L}\right)+K\left(L_{\infty }-\bar{L}\right)}{Z\left(L_{\lambda }-\bar{L}\right)+K\left(L_{\infty }-\bar{L}\right)}\;,$$ (3)

where *K* and *L*_*∞*_ are parameters of the von Bertalanffy growth equation (assumed to be constant over time), *L*_*c*_ is the mean size at first capture (obtained from the catch data), and *L*_*λ*_ is the expected size at oldest known age, respectively (see next section for more details on how these parameters were obtained). We selected Eq (3) instead of the Beverton-Holt model relating mean length to *Z* [23] because of a known bias in their model associated with the assumption of an infinite lifespan [19]. It is important to note that that Eqs (1) and (3) are valid in equilibrium conditions where fishing mortality has been relatively constant for a sufficient amount of time for the population to be in a stable state. An analysis of temporal trends in average lengths suggested that this was the case for the species in the current study (see Results for further details). If this had not been the case, we would have had to rely on non-equilibrium models [24]. Fishing mortality was obtained from *F* = *Z*-*M*, where *M* is the instantaneous natural mortality rate which was derived from longevity in our study (see section on mortality rates below for more details).

### Data sources

Application of the average length mortality estimator (Eq 3) and analyses of fishery sustainability (see below) required two types of information: basic life history information and length composition data. Life history parameters for longevity, growth in length and weight, and size-at-reproductive maturity were obtained from a synthesis of the scientific literature (S1 Table). We found life history studies conducted in Hawaii for only 10 species and thus had to rely on studies from other tropical Pacific regions for the other 9 species. When size-at-maturity was different between sexes, female size-at-maturity was used.

Size composition data were obtained from two sources: (1) underwater diver-based visual surveys; and, (2) commercial fishery trip reports. Between 2007 and 2013, teams of highly trained divers from the NOAA Pacific Island Fisheries Science Center (PIFSC) and from the PMNM offices conducted 770 and 764 visual surveys throughout the MHI and NWHI, respectively. Survey sites around each island or atoll were randomly selected within strata defined by combinations of reef zone (i.e., forereef, backreef, and lagoon) and depth-bins (shallow, 0–6 m; mid, 6–18 m; and deep, 18–30 m). At each sample site, independent stationary point counts were conducted by two paired divers inside contiguous 15 m diameter cylinders that extended from the bottom to the surface [11,25,26]. A diver first recorded a list of fish species observed during an initial 5 minute period. The diver then worked through this list, species by species, recording counts and estimating sizes of all fish seen within the cylinder. Fish sizes were recorded as total lengths to the nearest cm. Fishes from species not listed during the initial 5 minute period, but observed later in the survey, were also recorded but classified in a different data category. Before participating in the surveys, divers were extensively trained in size estimation using fish cut-outs of various sizes. Diver performance during and after survey efforts was evaluated by comparing size and count estimates between paired divers, where divers were continuously rotated between teams. Permits to conduct field surveys were obtained from the Hawaii Department of Land and Natural Resources (MHI surveys) and the Papahanaumokuakea Marine National Monument authorities (NWHI surveys).

Commercial fisheries data from 2003 to 2012 were obtained from commercial trip reports submitted by fishers to the Hawaii Division of Aquatic Resources (DAR). Trip records were utilized from the 3 main fishing gears used in inshore areas: hook-and-line (44% of fishing trips for the species targeted in this study), spearfishing (40%), and various nets (15%). Other gears, such as traps, represented less than 1% of trips for the species selected in the current study and data from these trips were not used. All 3 main fishing gears had a similar selectivity pattern, as inferred from the similar size composition of their catch (i.e. similar L_c_ and no indication of lower selectivity at larger sizes). We therefore combined length observations from all 3 fishing gears. Since lengths were not directly reported, the catches in weight were divided by the number of fish caught to obtain average weights per species per individual trip over the total of 47,439 trip records. Average weights were then converted to total lengths using published standard allometric weight (*W*)-length (*L*) relationships (see S1 Table for sources),

$$L={\left(\frac{W}{\alpha }\right)}^{\left(\frac{1}{\beta }\right)}\;,$$ (4)

where *α* is a scaling and *β* is a volumetric model parameter. Average lengths were calculated across trips using numbers caught per trip as a weighting variable. There were some concerns that converting average weight per trip to average length per trip would lead to a biased estimate of average length (Jensen’s inequality caused by the non-linear length-weight relationship; [27]). However, for each species, we compared the average length calculated from trips with only 1 fish in the catch vs. the average calculated for all trips and did not find differences in average length. The resulting length observations for each trip were checked to ensure that no lengths were greater than the maximum reported for each species (this step was also done for the diver surveys).

Catch records for certain taxa that were not identified to the species level were not included in our analyses (e.g., parrotfishes and the “kala” group of surgeonfishes composed of *Naso unicornis*, *N*. *annulatus*, and *N*. *brevirostris*).

### Estimation of average length and mortality rates

Mean lengths were evaluated for the exploited size range (*L* ≥ *L*_*c*_). Size at first capture *L*_*c*_ was set as the minimum length at full exploitation based on examination of the length converted fishery-dependent data for the principal gears targeting Hawaiian reef fishes (hook-and-line, nets, and spearfishing). To do so, we looked for clear discontinuous breaks in the size composition histograms for the commercial dataset. For example, the commercial catch record for *Parupeneus porphyreus* did not have individuals below 20 cm, had a few individuals in the 20–22 cm and 22–24 cm ranges (~50), and a noticeably higher number of individuals in the 24–26 cm range (close to 200 fish). The number of fish in size bins above 24–26 cm increased steadily. This discontinuous jump between 22–24 and 24–26 cm appeared to be related to selectivity; consequently the *L*_*c*_ was set at 25 cm. Lengths below *L*_*c*_ were discarded for both the commercial data and diver survey when calculating $\bar{L}$.

Species-specific average lengths were estimated for two broad geographical regions, the MHI and the unexploited Northwestern Hawaiian Islands (Fig 1). $\bar{L}$ were estimated from the $\bar{L}$ in four subregions weighted by the respective size of their shallow reef areas (depth <18m; Table 1). This was done to account for differences in size composition due to uneven fishing pressure (inferred from humans per reef area values—Table 1) and uneven sampling effort between regions. Standard errors and 95% confidence intervals were calculated in a similar fashion, using reef area as a weighting factor. For two species not commonly found in the NWHI, *Cephalopholis argus* and *Scarus rubroviolaceus*, length observations were imported from two other pristine areas (Wake and Johnston atolls).

Estimates of total instantaneous mortality rates *Z* were computed from Eq (3) using a numerical procedure (Ault et al., 1996). Values of *L*_*λ*_ (expected length at maximum age *a*_*λ*_) were estimated from the von Bertalanffy growth function using an observed maximum age.

Natural mortality rates *M* were estimated from lifespan applying the procedure of Alagaraja [28] (also see [29,30]), but assumes that 5% of a cohort survives to the observed maximum age (instead of about 1.5% in these papers):

$$M=\frac{-\text{ln}(S)}{a_{\lambda }}\;,$$ (5)

where *S* is the cohort survivorship to the maximum observed age (*a*_*λ*_). We originally selected the 5% survivorship value to obtain a more conservative estimate of *M* than the one obtained with the 1.5% value. As an added precaution, we independently estimated *S* by using Eq (3) to obtain *Z* in the NWHI for each species and fitted a linear regression model between the estimates of *Z* and 1/*a*_*λ*_ (making the reasonable assumption that *Z* = *M* in the NWHI). To do so, we represented Eq (5) as *M* = *b*_1_*1/*a*_*λ*_ (no intercept parameter). The slope (*b*_*1*_) of this regression is–ln(*S*) from which we derived *S* (i.e. *S* = e^-b1^). Finally, fishing mortality *F* was obtained by subtracting *M* from *Z*. Natural mortality *M* was assumed constant for all ages, and *F* was assumed constant for ages above *a*_*c*_.

### Numerical population model and stock assessment

Stock assessment involved comparison of various population metrics at current levels of fishing mortality relative to standard fishery management sustainability benchmarks [13]. For exploited fish populations, reference points for sustainability risks (spawning potential ratio, SPR) and fishery yields were computed using a length-conditional-on-age structured numerical model (following Ault et al. 1998) to simulate exploited fish populations. The computations were based on the mortality rates derived from $\bar{L}$ estimates and life history parameters synthesized from the literature. Numerical abundance at age *a* was estimated through use of an exponential mortality function (Eq 2). Length at age was estimated from the von Bertalanffy growth equation, and converted to weight-at-age using the allometric weight-length relationship (Eq 4). As an initial check on the validity of the population model and life history parameters, the model-calculated expected average length with no fishing mortality (${\bar{L}}_{F=0}$) was compared with the observed average length in the relatively pristine NWHI region.

The numerical model was used to obtain an important measure of stock reproductive potential, spawning stock biomass (SSB), at given levels of fishing mortality by summing over individuals in the population between the ages of sexual maturity (*a*_*m*_; age where 50% of individuals are mature, with knife-edge assumption) and oldest age *a*_λ,_

$$SSB=\sum_{a_{m}}^{a_{\lambda }}N(a)W(a)\;,$$ (6)

where *N(a)* is the average abundance at age *a* and *W(a)* is the mean weight of individuals at age *a*. Theoretically, a stock’s maximum reproductive biomass occurs when there is no fishing. Spawning potential ratio (SPR) is a management benchmark that measures a stock’s potential to produce yields on a sustainable basis. It was computed as the ratio of the current SSB relative to that of an unexploited stock

$$SPR=\frac{SSB_{\text{exp}\;loited}}{SSB_{un\;\text{exp}\;loited}}\;.$$ (7)

**Fig 2:**
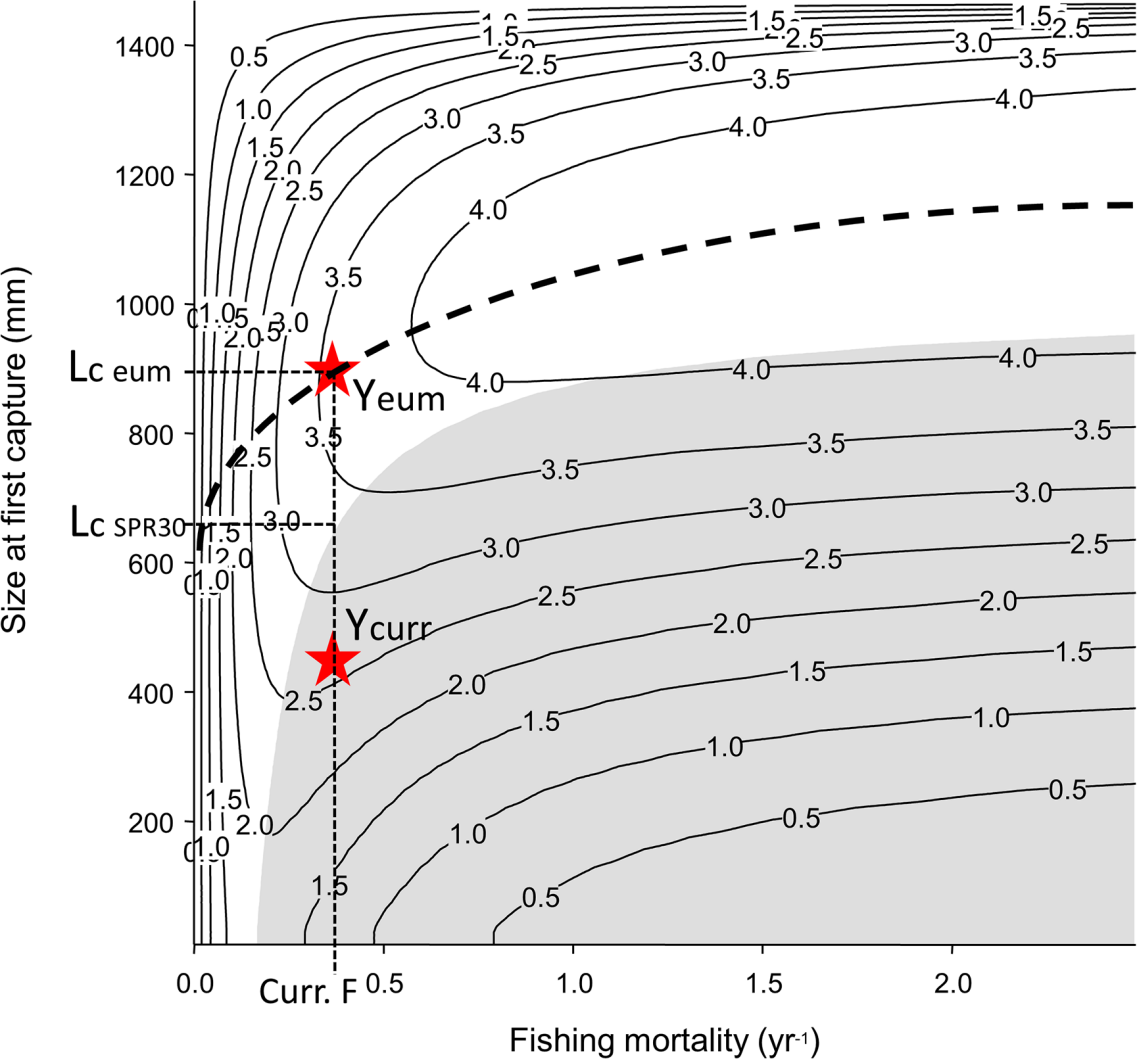
Example of yield-per-recruit isopleths. Yield as a function of fishing mortality rates (F) and sizes at first capture (*L*_*c*_) for the giant trevally *Caranx ignobilis*. *Y*_curr_ represents current yield-per-recruit (in kg) in the fishery and *Y*_eum_ is the highest possible yield for the current *F* (0.4). The gray area represents combinations of *F* and *L*_*c*_ that result in SPRs below 30%. *L*_*c*_ eum is the minimum size limit that will maximize yield while *L*_*c*_ SPR30 is the minimum size limit that will lead to an SPR of 30% given the current *F*.

Estimated SPRs were compared to USA federal standards which define 30% SPR as the threshold below which a stock is no longer sustainable (i.e., is experiencing recruitment overfishing) a standard recommended for less well-known stocks [31–33]. An associated reference point, the average length when SPR is equal to 30% (${\bar{L}}_{SPR\;30}$), was also evaluated as well as *L*_c SPR30_, the size at first capture required to obtain an SPR = 30% for current fishing mortality rates (Fig 2).

The numerical population model was also used to examine growth overfishing, which occurs when fish are caught before they have reached their full yield potential, thus limiting the production of harvested fish biomass [22]. The population metric yield per recruit (YPR) was calculated as

$$YPR=\frac{1}{R}\sum_{a_{c}}^{a_{\lambda }}FN\;(a)W\;(a)\;,$$ (8)

where *R* represents a fixed number of annual recruits (i.e., this number does not influence YPR values since yield is standardized to a single recruit), *F* is the fishing mortality rate, and *a*_*c*_ is the age of recruitment to the fishery (i.e., age at first capture). As a measure of growth overfishing, we calculated the expected percent increase in YPR from the current YPR associated with increasing the size at first capture (*L*_*c*_) to its eumetric value (i.e., *L*_*c eum*_ = *L*_*c*_ that maximizes YPR for the current *F*; see Fig 2).

## Results

### Average length and mortality estimates

**Fig 3:**
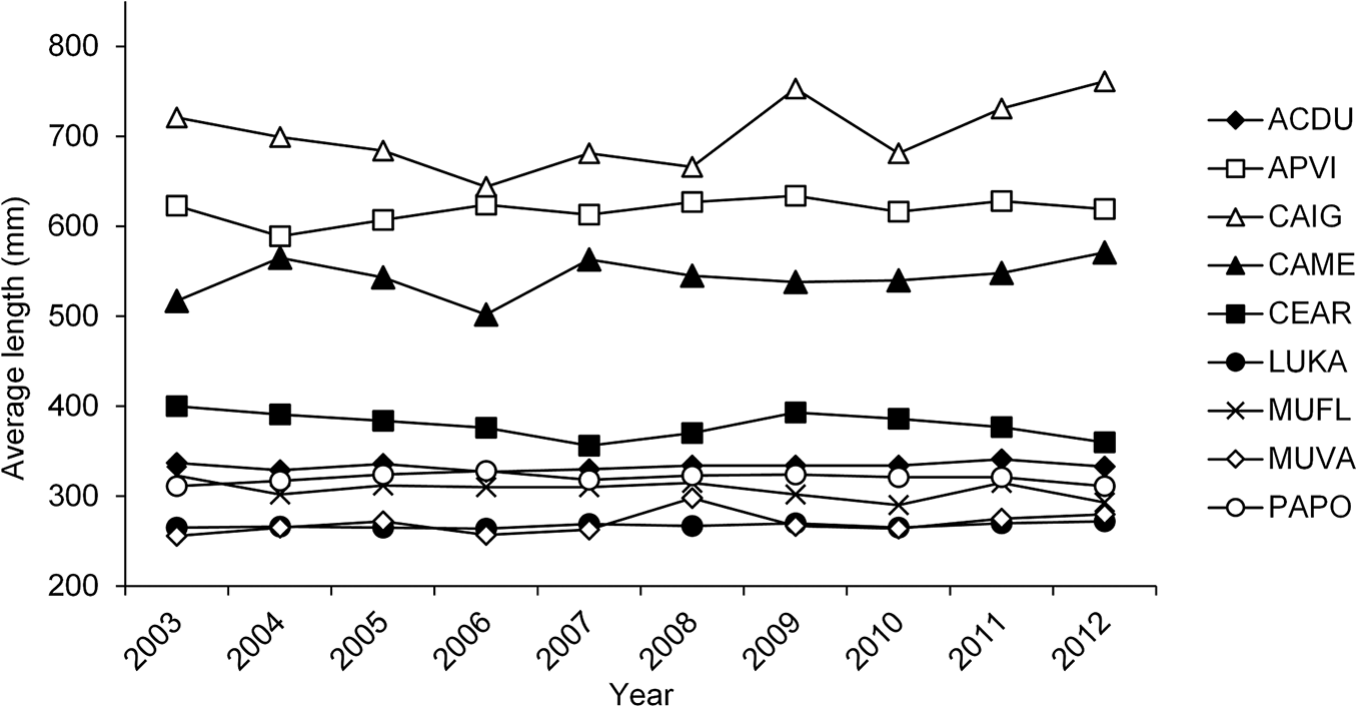
Time-series of average lengths in the exploited phase of the population. Average lengths displayed for 9 Hawaiian reef fish species in the MHI from 2003 to 2012. Species included in this analysis had at least 30 length observations per year. Data from commercial fishery reports. Species codes are defined in Table 2.

Length composition data (Table 2) and life history information (Table 3) were available for 19 Hawaiian reef fish species: three parrotfishes, six surgeonfishes, three goatfishes, two snappers, three jacks, one grouper, and one squirrelfish. Since our length-based mortality model assumed equilibrium conditions, we first plotted average length as a function of time (Fig 3). Annual estimates of $\bar{L}$ in the main Hawaiian Islands for the period 2003–2012 (commercial data) did not show any increasing or decreasing trends for 9 species with sufficient annual observations (Fig 3; n_yr_ > 30). This suggested that these stocks were basically at equilibrium. Although we did not have sufficient observations to conduct similar analyses for the other 10 species in our study, we assumed these were also at equilibrium given that all 9 of the other species were. Length observations were subsequently combined across years by data source (commercial, underwater diver surveys) to increase the precision of average length estimates.

**Table 2:** Average size ($\bar{L})$ and standard error (TL mm) for 19 exploited Hawaiian reef fishes in the 4 main Hawaiian Islands subregions and Northwestern Hawaiian Islands (NWHI) region. Only $\bar{L}$ estimates with a minimum of n = 10 observations are presented. The simulated average lengths when F = 0 from our population model are also presented (Sim. Ref., $\bar{L}$_F = 0_).

Species	Code	Data source^a^	Hawaii		Maui Nui		Oahu		Kauai—Niihau		NWHI		Sim. ref.
			n	$\bar{L}$	n	$\bar{L}$	n	$\bar{L}$	n	$\bar{L}$	n	$\bar{L}$	$\bar{L}$_F = 0_
*Chlorurus spilurus*–P^b^	CHSP	UVS	96	277(6)	241	241(3)	-	-	-	-	853	249(2)	249
*Scarus psittacus*–P	SCPS	UVS	35	269(5)	121	252(3)	10	266(12)	14	262(6)	117	262(3)	260
*Scarus rubroviolaceus*—P	SCRU	UVS	141	379(7)	217	408(7)	15	445(22)	176	405(9)	47	401(16)^c^	459
*Acanthurus blochii*–S	ACBL	UVS	98	301(3)	101	290(4)	-	-	92	303(4)	203	333(4)	332
*Acanthurus dussumieri*—S	ACDU	UVS	256	323(2)	269	310(2)	-	-	150	323(3)	142	330(4)	339
		COM	643	334(1)	731	311(2)	2507	340(1)	103	344(4)	-	-	“
*Naso brevirostris*–S	NABR	UVS	41	271(6)	192	255(2)	25	251(7)	41	346(11)	303	289(4)	300
*Naso lituratus*–S	NALI	UVS	277	277(2)	384	280(2)	33	268(6)	186	285(3)	368	295(2)	296
		COM	233	289(2)	297	289(2)	210	291(3)	-	-	-	-	“
*Naso hexacanthus*–S	NAHE	COM	180	466(3)	82	528(5)	196	512(3)	-	-	-	-	551
*Naso unicornis*–S	NAUN	UVS	37	344(10)	103	356(6)	10	337(18)	100	347(6)	2107	386(2)	445
*Mulloidichthys flavolineatus*—G	MUFL	UVS	-	-	94	283(2)	16	355(4)	24	302(4)	124	305(3)	312
		COM	207	313(3)	253	299(2)	757	304(1)	14	291(8)	-	-	“
*Mulloidichthys vanicolensis*—G	MUVA	UVS	67	236(4)	318	229(1)	33	269(6)	43	273(5)	342	251(2)	246
		COM	199	268(2)	75	278(4)	688	257(1)	125	266(3)	-	-	“
*Parupeneus porphyreus–*G	PAPO	COM	1071	334(1)	259	326(2)	1709	294(1)	264	330(2)	-	-	392
*Aprion virescens*–Sn	APVI	UVS	15	579(22)	57	601(19)	-	-	80	604(13)	1042	682(5)	705
		COM	645	611(3)	1380	603(2)	738	600(3)	937	684(3)	-	-	“
*Lutjanus kasmira*–Sn	LUKA	UVS	164	275(2)	208	260(2)	-	-	363	262(1)	367	273(2)	275
		COM	1511	264(1)	617	265(1)	1047	268(1)	560	270(1)	-	-	“
*Caranx melampygus*—J	CAME	UVS	11	433(17)	-	-	-	-	42	517(14)	858	575(5)	566
		COM	331	545(6)	485	538(5)	719	544(4)	563	552(5)	-	-	“
*Caranx ignobilis*–J	CAIG	UVS	-	-	-	-	-	-	-	-	1397	886(5)	856
		COM	140	769(21)	355	675(10)	923	678(6)	309	746(12)	-	-	“
*Seriola dumerili*–J	SEDU	COM	44	740(26)	56	827(29)	189	722(13)	141	793(12)	-	-	861
*Cephalopholis argus—*Gr	CEAR	UVS	96	380(5)	128	380(4)	-	-	63	390(6)	90	377(5)^c^	374
		COM	89	409(5)	664	364(1)	989	359(1)	38	391(10)	-	-	“
*Myripristis berndti*—So	MYBE	UVS	154	222(3)	88	212(3)	11	233(8)	207	224(3)	354	224(2)	229

^*a*^ *UVC*, *underwater visual census; COM*, *commercial report*.

^*b*^ *Family*: *P*, *parrotfishes (Labridae); S*, *surgeonfishes (Acanthuridae); G*, *goatfishes (Mullidae); Sn*, *snappers (Lutjanidae); J*, *jacks (Carangidae); Gr*, *groupers (Epinephelidae); Sq*, *squirrelfishes (Holocentridae)*.

^*c*^ *Also includes data from Johnston and Wake Atoll*.

**Table 3:** Life history parameters, mortality rates, and sustainability benchmarks for 19 Hawaiian reef fishes. See text for description of life history parameters and symbols used. Only species with at least 30 length observations were analyzed. Potential yield increase is the increase in yield that would result if fishing is eumetric (L_c_ = L_c_ eumetric). Lc SPR30 is the minimum size at full selectivity for SPR to be equal to 30% under current fishing mortality rate (F). See the S1 Table for life history parameter references.

Species	*L*_*∞*_ *(mm)*	*K (y* ^*-1*^ *)*	*a*_*0*_ *(y)*	*L*_*m*_ *(mm)*	*α*	*β*	*L*_*c*_ *(mm)*	*L*_*λ*_ *(mm)*	*a*_*λ*_ *(y)*	*M (y* ^*-1*^ *)*	$\bar{L}$ *(mm)*	*F (y* ^*-1*^ *)*	SPR (%)	Potential yield increase *(%)*	*L*_*c*_ *SPR30* (mm)	*L*_*c*_eumetric (*mm*)
**Parrotfishes**																
Chlorurus spilurus*	289	0.44	-0.76	170	0.024	2.97	200	287	10	0.30	253	0.01	99	-	-	-
Scarus psittacus*	278	1.65	-0.29	196	0.010	3.32	210	278	6	0.50	258	0.16	77	3	1	161
*Scarus rubroviolaceus* *	563	0.29	-0.81	374	0.014	3.11	260	562	22	0.14	401	0.20	27	13	310	405
**Surgeonfishes**																
Acanthurus blochii*	363	0.25	-0.38	276	0.025	3.03	250	363	35	0.09	297	0.27	16	6	305	296
Acanthurus dussumieri*	371	0.30	-0.29	282	0.025	3.03	260	371	28	0.11	317	0.18	32	1	251	279
Naso brevirostris*	327	0.40	-0.21	269	0.011	3.24	220	327	25	0.12	288	0.11	45	1	1	236
Naso lituratus*	322	0.34	-0.66	250	0.050	2.84	230	322	25	0.12	277	0.21	30	1	228	242
*Naso hexacanthus*	599	0.22	-0.22	511	0.042	2.85	410	599	44	0.07	509	0.13	23	2	480	461
Naso unicornis*	512	0.17	-0.50	330	0.018	3.04	260	512	53	0.06	350	0.25	8	44	436	430
**Goatfishes**																
*Mulloidichthys flavolineatus*	371	0.56	-0.36	199	0.009	3.21	260	364	6	0.50	301	0.42	61	5	1	220
Mulloidichthys vanicolensis*	267	1.3	-1.1	206	0.017	2.96	210	267	5	0.60	251	0.01	99	-	-	-
*Parupeneus porphyreus*	547	0.54	-0.45	264	0.011	3.21	250	530	6	0.50	318	1.32	14	28	349	382
**Snappers**																
*Aprion virescens*	810	0.31	0	500	0.022	2.89	450	810	26	0.12	621	0.23	23	10	553	623
*Lutjanus kasmira*	340	0.29	-1.37	200	0.008	3.25	240	318	8	0.37	267	0.36	63	12	146	190
**Jacks**																
*Caranx melampygus*	1041	0.23	-0.04	475	0.024	2.94	370	839	7	0.43	544	0.16	65	1	1	394
*Caranx ignobilis*	2170	0.11	0.10	839	0.015	3.09	430	1523	11	0.27	707	0.30	21	36	639	902
*Seriola dumerili*	1272	0.23	-0.79	910	0.024	2.86	450	1237	15	0.20	773	0.14	36	6	1	681
**Other families**																
*Cephalopholis argus*	506	0.08	-6.50	268	0.020	2.99	310	458	25	0.12	376	0.01	99	-	-	-
Myripristis berndti*	271	0.15	-4.48	175	0.028	3.00	180	268	27	0.11	224	0.04	69	5	1	130

* UVC data used for average length. *All other species*, *commercial data used*.

**Fig 4:**
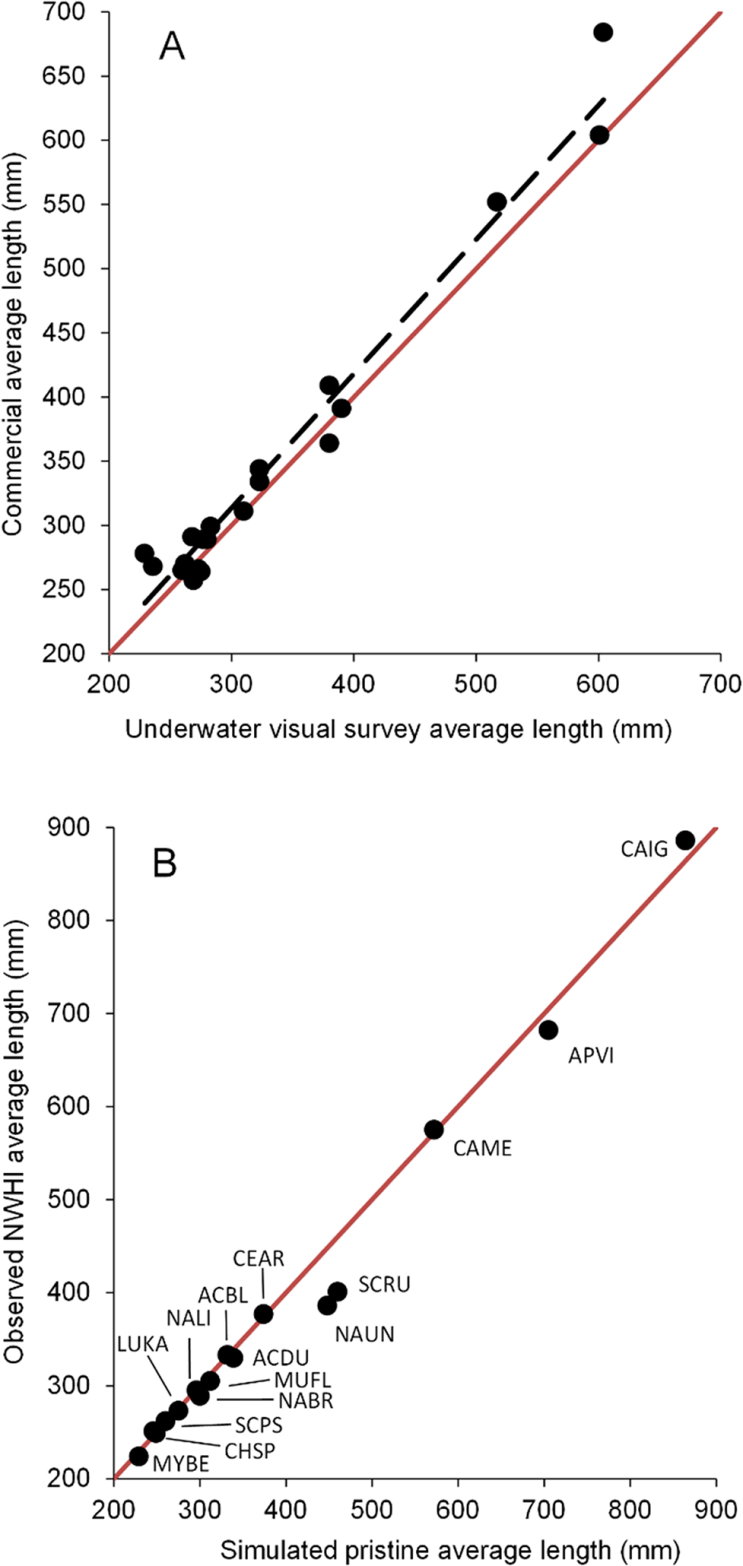
Average length comparisons. (A) Comparison of average lengths in the commercial dataset versus the underwater visual survey dataset for the main Hawaiian Islands (MHI). Closed circles represent average lengths by species in different subregions of the MHI. (B) Average lengths observed in the Northwestern Hawaiian Islands versus simulated unexploited (pristine) average lengths. The red line represents perfect agreement between the two sets of average lengths. Species codes are defined in Table 2.

Table 2 shows average lengths by species and subregion. For most species, there was no clear pattern in average length between the more densely populated MHI subregions (Maui Nui and Oahu) and the less populated ones (Hawaii and Kauai-Niihau) (Table 2). Notable exceptions were *Parupeneus porphyreus*, *Aprion virescens*, and *Caranx ignobilis* which showed clearer patterns of decreasing average lengths (and thus increasing *F*s) across a human population gradient (Tables 1 and 2). This was particularly the case for *C*. *ignobilis*, which showed distinct differences in average length between Hawaii and Kauai-Niihau (758 mm) and Maui Nui and Oahu (677 mm; the latter island having by far the most densely populated coastline). Average lengths for the commercial dataset were generally in agreement with those from underwater surveys (Fig 4A). Some exceptions were *C*. *melampygus*, which may be due to a low numbers of observations in underwater surveys. For each species, we selected the data source with the most length observations for subsequent analyses.

**Fig 5:**
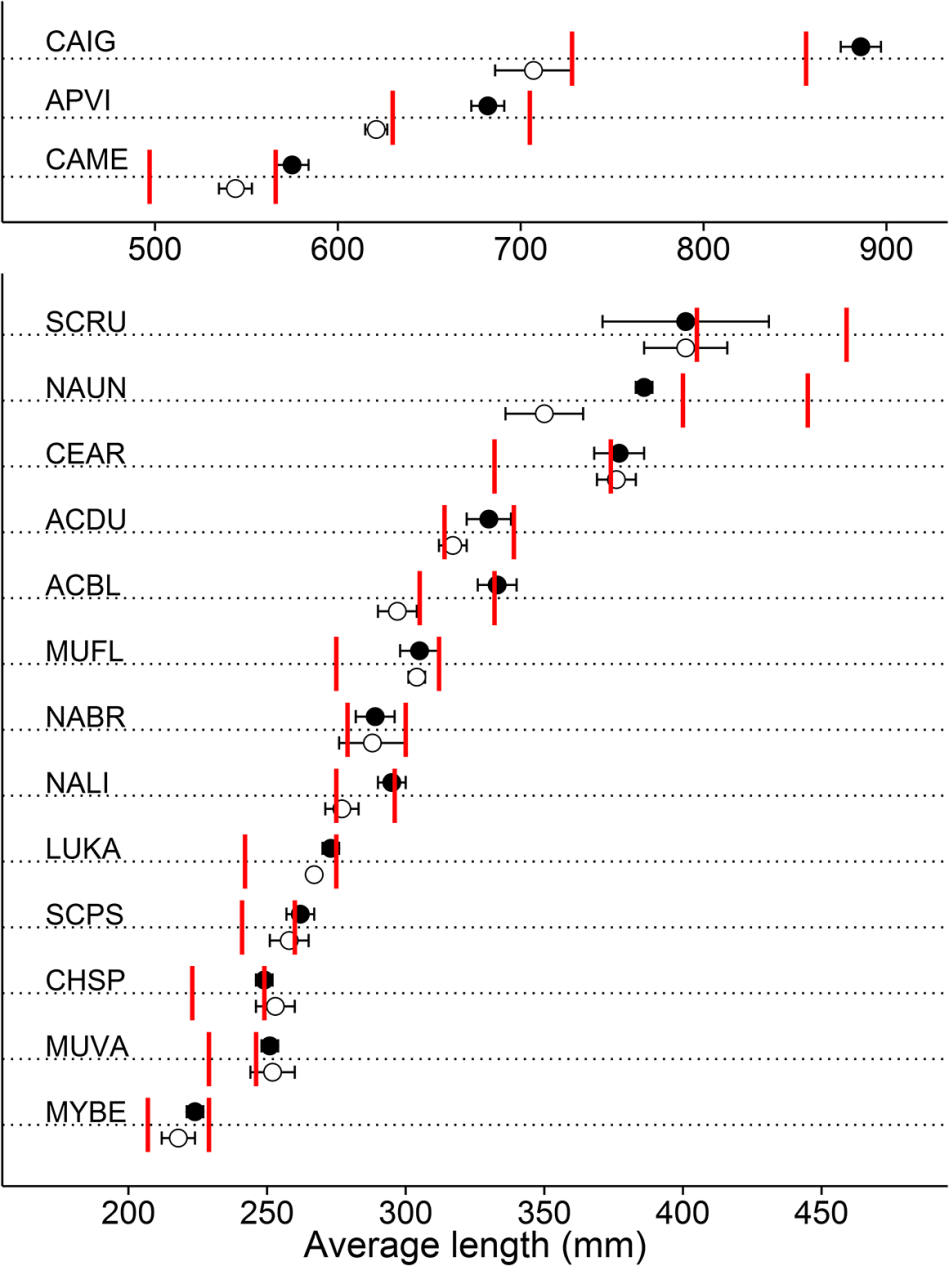
Average lengths ($\bar{L}$) and 95% CIs for the main Hawaiian Islands (MHI; open circles) and the Northwestern Hawaiian Islands (NWHI; closed circles). Two reference points are also displayed: first red bar, ${\bar{L}}_{SPR\;30}$ (average length when SPR = 30%); second red bar, ${\bar{L}}_{F=0}$ (average length when F = 0). Species are ordered by maximum size. Only species with n>30 in the NWHI are presented. Species codes are defined in Table 2.

The average lengths of species in the pristine NWHI were either larger (11 species) or similar (5 species) relative to the inhabited MHI (Table 2). Furthermore, 10 species had an overlap between their $\bar{L}$ 95% confidence intervals in the NWHI and their corresponding simulated ${\bar{L}}_{F=0}$ (Fig 4B and Fig 5). The remaining 6 species had average lengths close to the expected value at *F* = 0, except for two species. The redlip parrotfish $\bar{L}$ estimate in the NWHI was likely not reliable due to few observations (n = 47), even after including data from Wake and Johnston Atoll. Surgeonfish *Naso unicornis* in the NWHI had average lengths smaller than expected (386 mm vs. 445 mm, respectively). This may be due to a low estimate of *M* (see Discussion section for more details).

**Fig 6:**
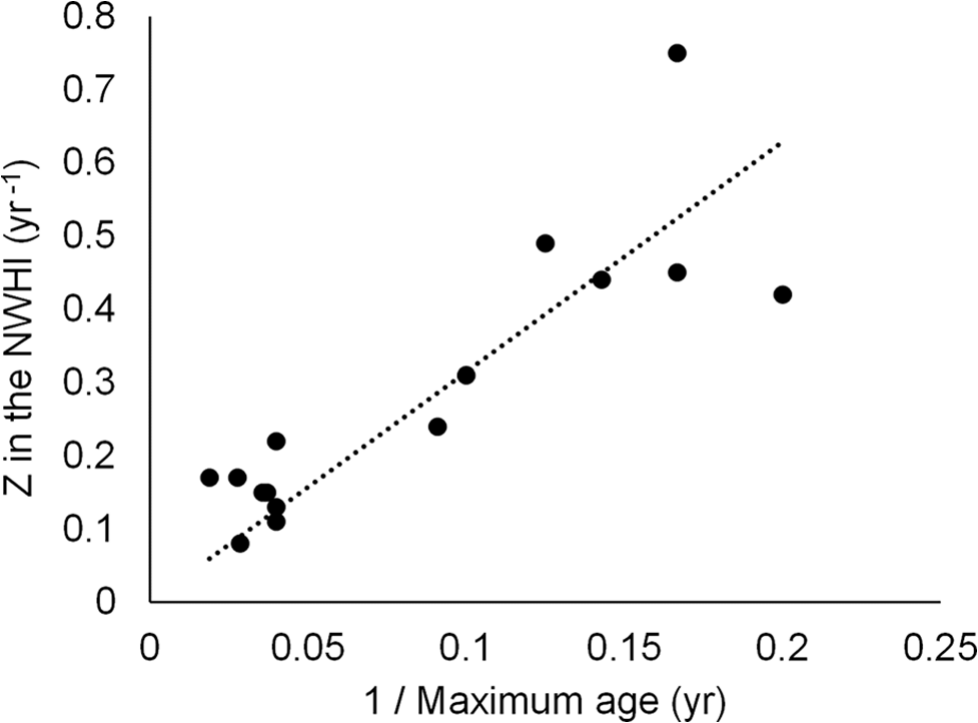
Linear regression of total instantaneous mortality rate Z in the NWHI vs. inversed of published maximum age. *Z* is derived from average length and is assumed to be equal to M. The slope of this regression is equal to–ln(*S*) = 3.14, where *S* (cohort survivorship to maximum age) is 0.043.

The regression of our *Z* estimates dependent on 1/*a*_*λ*_ for 15 species in the NWHI (where Z was assumed to be equal to *M*) gave a slope of–ln(*S*) = 3.143 (r^2^ = 0.71; Fig 6), which is equivalent to a survivorship to the maximum age *S* of 0.043 (i.e., from e^-3.143^). This value is very close to the *S* = 0.05 used in our analyses.

Estimated fishing mortality rates in the MHI region varied greatly by species, ranging from 0.01 to 1.32 (Table 3). The two species with the highest fishing mortality rates were the goatfishes *Parupeneus porphyreus* (*F* = 1.32) and *Mulloidichthys flavolineatus* (*F* = 0.46), followed by the snapper *Lutjanus kasmira* (*F* = 0.36), and the jack *Caranx ignobilis* (*F* = 0.30). The fishing mortality rates for all other species were less than 0.3.

### Stock assessment

**Fig 7:**
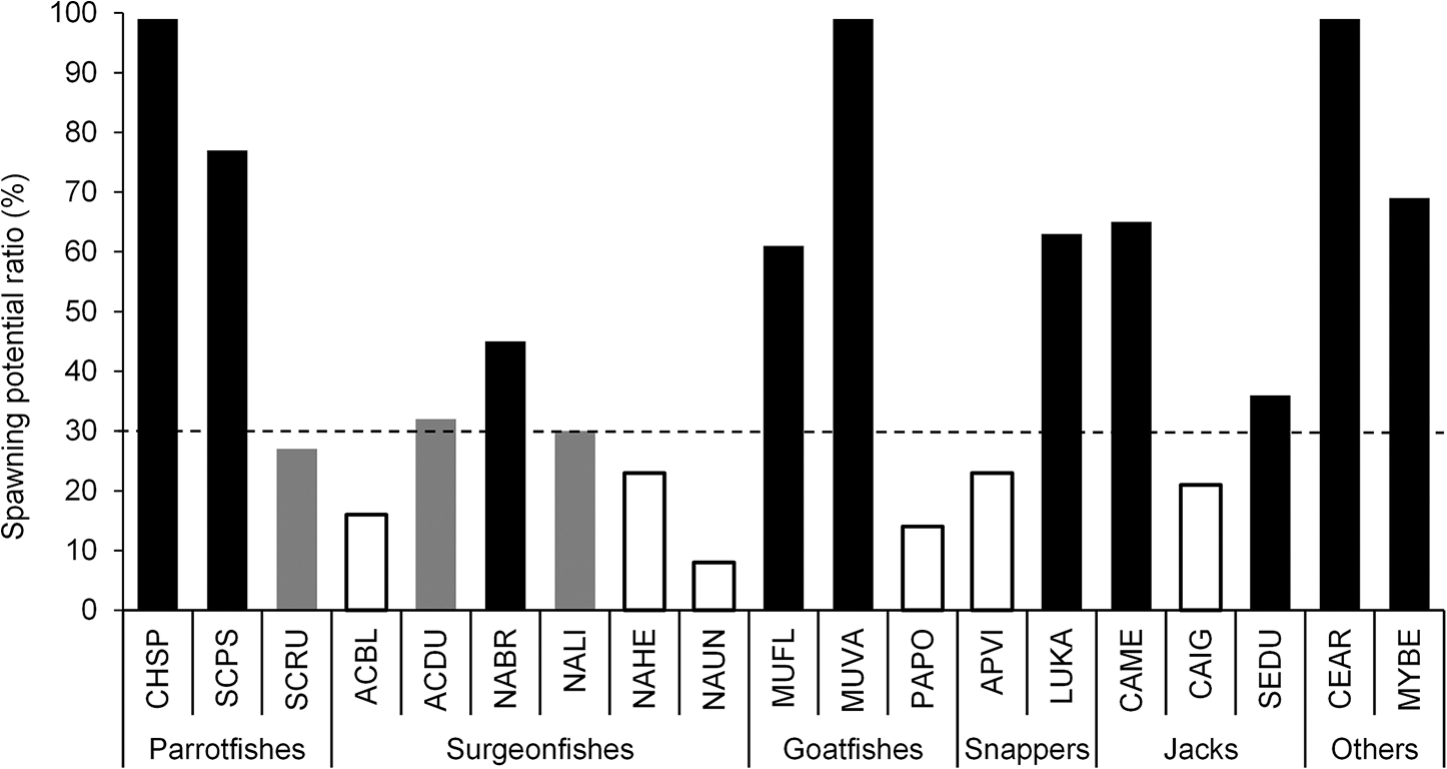
Spawning potential ratio (SPR) for 19 Hawaiian reef fishes in the main Hawaiian Islands. White bars, SPR < 25%; gray bars, SPR between 25% ≤ SPR ≤ 35%; black bars, SPR > 35%. Species codes are defined in Table 2. Dashed line denotes minimum SPR threshold of 30%.

Of the species evaluated in this study, 53% had SPR estimates above the lower limit of 30%, while 16% were between 25–35% SPR and 31% were below 25% SPR (Table 3; Fig 7). In general, the 95% confidence intervals for estimates of $\bar{L}$ were much smaller than the interval between ${\bar{L}}_{SPR30}$ and ${\bar{L}}_{F=0}$, suggesting there was sufficient contrast in our length estimates between lightly-fished or unfished areas versus heavily-fished areas to draw meaningful conclusions on stock status (Fig 5). SPR estimates were generally higher for parrotfishes (average SPR of 68%), goatfishes (58%), and jacks (41%), while surgeonfishes had generally low SPRs (26%). The two invasive species analyzed, (*Cephalopholis argus* and *Lutjanus kasmira*), had relatively high SPRs (> 60%).

Potential yield increases (from increasing *L*_*c*_ to its eumetric value), a measure of growth overfishing, was relatively low for most species (ranging between 1% and 44%; Table 3). Potential gains in yield exceeding 25% were found for *Naso unicornis*, *Caranx ignobilis*, and *Parupeneus porphyreus*.

## Discussion

The sustainability of coral reef fisheries has been questioned [34,35], but this conjecture has generally been based on heuristic evidence such as observed differences in relative abundance between fished and protected or uninhabited areas [11,36–38]. There have been few formal stock assessments of tropical coral reef fishes other than those conducted in Florida, Puerto Rico, and more recently Belize [13,16,39,40]. The sustainability of reef fisheries in other regions, such as the tropical Pacific, has yet to be assessed. Our study presents the first major assessment of shallow water coral reef fish species in Hawaii. While other recent studies have had to rely on incomplete catch time-series and family-level analyses [17,18], we used a relatively simple and reliable [19,24,41] assessment method with minimal data requirements to obtain the mortality rates necessary to run population dynamic models at the individual species level. We show that more than half of the exploited species analyzed in this study have a spawning potential ratio above the US federal standard of 30% and are likely not experiencing recruitment overfishing. A typical finding was that the species faring best were generally the shorter lifespans with higher natural mortality rates (e.g., the smaller parrotfishes *Chlorurus spilurus* and *Scarus psittacus*; some of the goatfishes; the jack *Caranx melampygus*; and the snapper *Lutjanus kasmira*). Species with lowest SPRs were generally the longer-lived, lower natural mortality species (e.g. the parrotfish *Scarus rubroviolaceus*; the snapper *Aprion virescens*, and surgeonfishes) or the more highly-prized goatfish *Parupeneus porphyreus* and jack *Caranx ignobilis*. These results are consistent with the well-established relationship between longevity and sensitivity of SPR to exploitation pressures, that is, longer-lived species have much of their pristine spawning biomass represented by older (larger) individuals whose numbers become significantly depleted even at relatively low fishing mortality rates [42].

Growth overfishing occurs when fish are harvested before they reach their optimum size (i.e., the size at which biomass loss to natural mortality equals the biomass gained from individual fish growth in a cohort), thus reducing fishery yields (i.e. harvested biomass). We examined the potential increases in yields if the sizes at first capture (*L*_*c*_) were set optimally for the estimated fishing mortality rates (i.e., eumetric fishing). For the majority of species in our study, there was little apparent gains in yields associated with adjusting L_c_ optimally, either because certain species had relatively low fishing mortality rates (e.g., *Myripristis berndti*, *Cephalopholis argus*, and *Chlorurus spilurus*) or had high individual growth rates accompanied by low natural mortality (i.e. maximum individual weights are reached quickly with little loss to natural mortality, thus reducing the influence of *L*_*c*_ in increasing yield; e.g. *Naso brevirostris*). The few species where large gains in yields could be achieved by adjusting L_c_ optimally were those experiencing higher fishing mortality rates (*F*>0.2) such as *Naso unicornis* (44% potential yield gain), *Caranx ignobilis* (36%) and *Parupeneus porphyreus* (28%).

It is important to note that some fish stocks in the densely populated Oahu subregion had lower average lengths, and thus higher fishing mortality rates and lower SPRs as compared to other less populated MHI subregions. For example, highly-prized goatfish *Parupeneus porphyreus* had SPRs outside of Oahu of around 20%, but for Oahu SPR was 6%. The jack *Caranx ignobilis*, also a popular recreational target, had an SPR of 16% for Maui Nui and Oahu, but much higher values around the lightly populated Hawaii and Kauai-Niihau (SPR >35%). Other species did not show significant differences in average lengths among subregions. This may have been due to either a low number of observations, well-mixed populations, or similar fishing mortality rates for these particular species among subregions. It is still not entirely clear to what level the fish populations around the MHI are connected, or if larval exchanges or adult movements exist between Oahu and the other Hawaiian Islands. A recent study failed to detect inter-island movements for tagged *Caranx ignobilis*, a large and highly mobile predator [43]. Genetic connectivity studies indicate that most reef fish species have no genetic structuring across the Hawaiian archipelago [44–46]. However, the absence of genetic structure does not necessarily imply that stocks are well connected at time scales relevant to the population dynamic processes. Population connectivity within the MHI is still an open question and will require further attention if potentially disconnected reef fish populations are to be managed sustainably, given the likely large differences in fishing pressures between subregions.

The reliability and robustness of conclusions regarding the state of Hawaiian reef fish populations depends on the veracity of a few assumptions. First, we assumed the reef fish stocks analyzed in the current study were at equilibrium (i.e., mortality rates and recruitment have been fairly constant over the last decade or so). Ault et al. (2005) showed that mortality rates derived from average length are fairly robust to even moderate levels of recruitment variation. In the case of an extreme recruitment event (e.g., an annual 10-fold increase in the background recruitment level), we would have expected average lengths to decrease dramatically for a few years followed by a quick upward rebound before a return to the long-term equilibrium. In the case of a long-term increasing trend in fishing mortality, we would have expected a slow, constant decline in average length. We did not observe such patterns in average length over time in our study and this suggests that potential fluctuations in recruitment levels over time were not significant enough to affect our average length estimates and that fishing mortality was more or less constant. It is of course possible that a long-term decrease in recruitment and a long-term increase in fishing mortality produced stable average lengths for the species in our study, but this seems unlikely.

Another key assumption was that size composition data were representative of the true population around the MHI. Both the underwater visual survey and commercial trip report datasets had strengths and weaknesses. The underwater surveys by SCUBA divers did not reach depths beyond 30 m due to safety and time constraints, but were able to sample remote and exposed areas of the MHI that are likely visited less frequently by fishers. The size composition data for the visual survey dataset was thus more representative of nearshore (<30m deep) communities but encompassed the entire nearshore waters in the MHI, including remote, lightly-populated, and relatively inaccessible sections of coastlines. On the other hand, size composition data from commercial trip reports included information on deeper fish communities, but were less likely to be representative of inaccessible coastlines. Despite these potential biases, the size composition information from these two disparate datasets was remarkably similar suggesting that the average lengths used in the current study were likely representative of the real values. Another important assumption was that the life history parameters used in our analyses were representative of the local fish populations in Hawaii. For many species, we had to use life history parameters from other Pacific areas. It is possible that these values change geographically and with environmental conditions ([47,48], although see [49]). The availability of an extensive underwater visual survey dataset for the relatively pristine NWHI allowed us to evaluate the validity of the length-based mortality model used in this study [16,19] as well as the validity of our life history information. We first used our independent estimates of *M* from the NWHI (where *Z* derived from average length is assumed to be equal to *M*) to derive an estimate of average cohort survivorship (*S*) to maximum age (*a*_*λ*_). We obtained a value closer to the one used for our analysis (0.043 vs. 0.05) than the one suggested elsewhere (0.015) [30,50]. The *S* = 0.015 value comes from empirical data that include mollusks, fish, and cetaceans, and thus may not be suitable for reef fishes. The exact survivorship value is linked to the sampling effort in the dataset from which a_λ_ is obtained (i.e. the larger the number of aged individuals, the greater the chance of finding extremely old individuals that are not representative of a 5% or even 1% cohort survivorship value) [51]. Since the a_λ_ value for reef fishes generally comes from life history studies with less than 100 aged individuals, it is possible that these values represent cohort survivorship higher than 1.5%, which is what our analysis suggests.

Additionally, we used the length-mortality model to generate expected average length when only natural mortality is present (*F* = 0), and we were able to compare these average lengths with observed values in the NWHI. For most species, the expected average lengths at *F* = 0 fell within the 95% confidence intervals of the observed average lengths, while average lengths of the remaining species were within a few centimeters of predicted values. One exception was the parrotfish *Scarus rubroviolaceus* which may have been due to a small sample size in the NWHI. Another clear exception was the surgeonfish *Naso unicornis* which had an average length in the NWHI much smaller than the predicted pristine average (386 mm vs. 445 mm). A likely reason for this discrepancy is the natural mortality estimate derived from longevity (53 years) may be overestimated by the assumption that 5% of a cohort is left at that age. This longevity estimate comes from an extensive ageing effort in the MHI and NWHI ([52]; n = 186) combined with an unpublished bomb radiocarbon dating analysis which confirmed that the oldest aged individual was 53 years (A. Andrews–NOAA Pacific Islands Fisheries Science Center, personal communication). If we used the average length in the NWHI in Eq (3) we would have obtained an *M* = 0.16, which would suggest a shorter lifespan. However, even with this less conservative natural mortality estimates, the SPR for this species remains close to the 30% SPR limit at 36% (up from 8%). With the exception of these two species, the convergence between the expected average length when *F* = 0 and the actual average length in the NWHI suggests that our length-mortality model, life history parameters, and size composition data were appropriate, and this strengthens our confidence in our conclusions concerning the state of reef fish stocks in Hawaii.

The coral reef fishes of Hawaii appear to be in slightly better shape as compared to other coral reef ecosystems within U.S. jurisdiction (i.e., Puerto Rico and Florida). In the current study, about 47% of the targeted species analyzed may be overfished (SPR < 30%). Studies elsewhere have shown a greater level of overfishing, e.g., in the Florida Keys (63% of species [16,39]) and Puerto Rico (70% of species [13]). The lower level of overfishing in Hawaii may reflect the relative inaccessibility of much of the coastline [9] and the concentration of most of the human population on a single island (70% on Oahu).

### Future directions

The data-poor length-based assessment approach used in the current study has now been used for the assessment of reef fish populations in Florida, Puerto Rico, and Hawaii, and we plan to extend the current effort to other Pacific areas, such as American Samoa and the Mariana archipelago. The simple data requirements associated with this approach make it readily exportable to other jurisdictions as well, especially in species-rich tropical countries with limited management budgets. There is, however, a clear need for more focused life history studies for the main exploited reef species in Hawaii and elsewhere. We currently often have to rely on growth and maturity information from geographically distinct regions, which may introduce some bias in our assessments. Having local length, age, and maturity raw data would also allow the integration of parameter uncertainty into future assessments instead of having to rely on a mostly deterministic approach, as in the current study.

## Supporting Information

S1 Table Source of life history parameters for the 19 Hawaiian reef fish species. (DOCX)
