# Supplementary material for: Length-Based Assessment of Coral Reef Fish Populations in the Main and Northwestern Hawaiian Islands
Source: PLoS One. 2015 Aug 12;10(8):e0133960. doi: 10.1371/journal.pone.0133960 (PMC4534412; doi:10.1371/journal.pone.0133960)
Supplement: S1 Table — (DOCX) [file pone.0133960.s001.docx]

S1 Table – **Source of life history parameters for the 19 Hawaiian reef fish species**

| Species | Code | Longevity (a*_λ_*) | Growth rate (Linf, K, a_0_) | Maturity (*L_m_)* | Length-weight parameters  (α, β) |
| --- | --- | --- | --- | --- | --- |
| **Parrotfish (Scaridae)** |  |  |  |  |  |
| *Chlorurus spilurus* | CHSP | (Choat et al., 1996) | (Page, 1998) | (Page, 1998) | (Kulbicki et al., 2005) |
| *Scarus psittacus* | SCPS | (Taylor and Choat, 2014) | (Page, 1998) | (Page, 1998) | (Kulbicki et al., 2005) |
| *Scarus rubroviolaceus* | SCRU | (Howard, 2008) | (Howard, 2008) | (Howard, 2008) | (Smith and Dalzell, 1993) |
|  |  |  |  |  |  |
| **Surgeonfish (Acanthuridae)** |  |  |  |  |  |
| *Acanthurus blochii* | ACBL | (Choat and Robertson, 2002) | (Choat and Robertson, 2002) | (Choat and Robertson, 2002)^a^ | (Kulbicki et al., 2005) |
| *Acanthurus dussumieri* | ACDU | (Choat and Robertson, 2002) | (Choat and Robertson, 2002) | (Choat and Robertson, 2002)^a^ | (Kulbicki et al., 2005) |
| *Naso brevirostris* | NABR | (Choat and Robertson, 2002) | (Choat and Robertson, 2002) | (Choat and Robertson, 2002) | (Kulbicki et al., 2005) |
| *Naso lituratus* | NALI | Unpublished^b^ | Unpublished^b^ | Unpublished^b^ | (Smith and Dalzell, 1993) |
| *Naso hexacanthus* | NAHE | (Choat and Robertson, 2002) | (Choat and Robertson, 2002) | (Choat and Robertson, 2002) | (Choat and Axe, 1996) |
| *Naso unicornis* | NAUN | Allen Andrews p. comm. | (Eble et al., 2009) | (Eble et al., 2009) ^c^ | (Kulbicki et al., 2005) |
|  |  |  |  |  |  |
| **Goatfish (Mullidae)** |  |  |  |  |  |
| *Mulloidichthys flavolineatus* | MUFL | Estimated ^d^ | (Holland et al., 1993) | (Cole, 2009) | (Holland et al., 1993) |
| *Mulloidichthys vanicolensis* | MUVA | (Cole, 2009) | (Cole, 2009) | (Cole, 2009) | (Jehangeer, 2003) |
| *Parupeneus porphyreus* | PAPO | (Moffitt, 1979) | (Moffitt, 1979) | (Moffitt, 1979) ^e^ | (Kulbicki et al., 2005) ^f^ |
|  |  |  |  |  |  |
| **Snappers (Lutjanidae)** |  |  |  |  |  |
| *Aprion virescens* | APVI | (Loubens, 1980) | (Loubens, 1980) | (Everson et al., 1989) | (Kulbicki et al., 2005) |
| *Lutjanus kasmira* | LUKA | (Loubens, 1980) | (Morales-Nin and Ralston, 1990) | (Allen, 1985) | (Kulbicki et al., 2005) |
|  |  |  |  |  |  |
| **Jacks (Carangidae)** |  |  |  |  |  |
| *Caranx melampygus* | CAME | (Fry et al., 2006) | (Sudekum et al., 1991) | (Sudekum et al., 1991) | (Seki, 1986) |
| *Caranx ignobilis* | CAIG | (Fry et al., 2006) | (Sudekum et al., 1991) | (Sudekum et al., 1991) | (Kulbicki et al., 2005) |
| *Seriola dumerili* | SEDU | (Manooch III and Potts, 1997) | (Manooch III and Potts, 1997) | (Kožul et al., 2001) | (Manooch III and Potts, 1997) |
|  |  |  |  |  |  |
| **Other families** |  |  |  |  |  |
| *Cephalopholis argus* | CEAR | (Donovan et al., 2012) | (Donovan et al., 2012) | (Myers,, 1999) | (Kulbicki et al., 2005) |
| *Myripristis berndti* | MYBE | (Craig and Franklin, 2008) | (Craig and Franklin, 2008) | (Murty, 2002) ^g^ | (Kulbicki et al., 2005) |

a Length at maturity is set at 76% of Linf (average lmat/linf ratio for surgeonfishes).

b Data from specimens collected by the NOAA Pacific Islands Fisheries Science Center in the Northern Marianas in 2009. Processing and analyses carried out at James Cook University.

c Size-at-maturity is average between males and females.

d No longevity estimate. Best estimate of longevity based on similar goatfish in Hawaii (*Mulloidichthys vanicolensis* and *Parupeneus porphyreus*).

e Size at first reproduction

f Used relationship for *Parupeneus multifasciatus*

g Size at maturity taken for a similar species, *M. murdjan*

# Literature cited

Allen, G.R. (1985). Snappers of the World: An Annotated and Illustrated Catalogue of Lutjanid Species Known to Date (Food and Agriculture Organization of the United Nations).

Choat, J.H., and Axe, L.M. (1996). Growth and longevity in acanthurid fishes; an analysis of otolith increments. Mar. Ecol. Prog. Ser. Oldendorf *134*, 15–26.

Choat, J.H., and Robertson, D.R. (2002). Age-based studies on coral reef fishes. In Coral Reef Fishes: Dynamics and Diversity in a Complex Ecosystem, (San Diego: Academic Press), pp. 57–80.

Choat, J.H., Axe, L.M., and Lou, D.C. (1996). Growth and longevity in fishes of the family Scaridae. Mar. Ecol. Prog. Ser. *145*, 33–41.

Cole, K.S. (2009). Size-dependent and age-based female fecundity and reproductive output for three Hawaiian goatfish (Family Mullidae) species, *Mulloidichthys flavolineatus* (yellowstripe goatfish), *M. vanicolensis* (yellowfin goatfish), and *Parupeneus porphyreus* (whitesaddle goatfish).

Craig, M.T., and Franklin, E.C. (2008). Life history of Hawaiian “redfish”: a survey of age and growth in “aweoweo (*Priacanthus meeki*) and u”u (*Myripristis berndti*) (Kaneohe, Hawaii: Hawaii Institute of Marine Biology).

Donovan, M.K., Friedlander, A.M., DeMartini, E.E., Donahue, M.J., and Williams, I.D. (2012). Demographic patterns in the peacock grouper (Cephalopholis argus), an introduced Hawaiian reef fish. Environ. Biol. Fishes.

Eble, J.A., Langston, R., and Bowen, B.W. (2009). Growth and reproduction of Hawaiian Kala, *Naso unicornis* (Honolulu, Hawaii: Fisheries Local Action Strategy, Division of Aquatic Resources).

Everson, A.R., Williams, H.A., and Ito, B.M. (1989). Maturation and reproduction in two Hawaiian eteline snappers, Uku, *Aprion virescens*, and Onaga, *Etelis coruscans*. Fish. Bull. *87*, 877–888.

Fry, G.C., Brewer, D.T., and Venables, W.N. (2006). Vulnerability of deepwater demersal fishes to commercial fishing: evidence from a study around a tropical volcanic seamount in Papua New Guinea. Fish. Res. *81*, 126–141.

Holland, K.N., Peterson, J.D., Lowe, C.G., and Wetherbee, B.M. (1993). Movements, distribution and growth rates of the white goatfish *Mulloidichthys flavolineatus* in a fisheries conservation zone. Bull. Mar. Sci. *52*, 982–992.

Howard, K.G. (2008). Community structure, life history, and movement patterns of parrotfishes: large protogynous fishery species (Honolulu: PhD thesis. University of Hawaii at Manoa).

Jehangeer, M.I. (2003). Some population parameters of the goatfish, Mulloidichthys vanicolensis from the lagoon of Mauritius (ACP-EU Fisheries Research Report (14)).

Kožul, Skaramuca, Kraljević, Dulčić, and Glamuzina (2001). Age, growth and mortality of the Mediterranean amberjack Seriola dumerili (Risso 1810) from the south-eastern Adriatic Sea. J. Appl. Ichthyol. *17*, 134–141.

Kulbicki, M., Guillemot, N., and Amand, M. (2005). A general approach to length-weight relationships for New Caledonian lagoon fishes. Cybium *29*, 235–252.

Loubens, G. (1980). Biologie de quelques especes de poissons du lagon Neo-Caledonien. III. Croissance. In Cahiers de l’Indo-Pacifique Vol 2, (Paris: Cahiers de l’Indo-Pacifique Vol. 2), pp. 41–72.

Manooch III, C.S., and Potts, J.C. (1997). Age, growth, and mortality of Greater Amberjack, *Seriola Dumerili*, from the U.S. Gulf of Mexico Headboat Fishery. Bull. Mar. Sci. *61*, 671–683.

Moffitt, R.B. (1979). Age, growth, and reproduction of the kumu, *Parupeneus porphyreus* (Honolulu: PhD thesis. University of Hawaii at Manoa).

Morales-Nin, B., and Ralston, S. (1990). Age and growth of *Lutjanus kasmira* (Forskaal) in Hawaiian waters. J. Fish Biol. *36*, 191–203.

Murty, V.S. (2002). Marine Ornemental Fish Resources of Lakshadweep.

Myers,, R.F. (1999). Micronesian reef fishes: a comprehensive guide to the coral reef fishes of Micronesia (Guam: Coral Graphics).

Page, M. (1998). The biology, community structure, growth and artisanal catch of parrotfishes of American Samoa (Pago Pago, American Samoa: Department of Marine and Wildlife Resources).

Seki, M.P. (1986). Carangidae. In Fishery Atlas of the Northwestern Hawaiian Islands, (NOAA Tech. Rep. NMFS 38), pp. 86–87.

Smith, A., and Dalzell, P. (1993). Fisheries resources and management investigations in Woleai Atoll, Yap State, Federated States of Micronesia (Inshore Fish. Res. Proj., Tech. Doc., South Pacific Commission. Noumea, New Caledonia.).

Sudekum, A.E., Parrish, J.D., Radtke, R.L., and Ralston, S. (1991). Life history and ecology of large jacks in undisturbed, shallow, oceanic communities. Fish. Bull. *89*, 493–513.

Taylor, B.M., and Choat, J.H. (2014). Comparative demography of commercially important parrotfish species from Micronesia: Comparative demography of parrotfishes. J. Fish Biol. n/a–n/a.
